# Finding my tribe: a qualitative interview study of how people living with metastatic breast cancer perceive support groups

**DOI:** 10.1007/s11764-024-01639-7

**Published:** 2024-07-24

**Authors:** Grace M. Mackie, Frances Boyle, Sophie Lewis, Andrea L. Smith

**Affiliations:** 1 Faculty of Medicine and Health, University of Sydney, Camperdown, NSW Australia; 2 Mater Hospital, Sydney, NSW Australia; 3 The Daffodil Centre, University of Sydney, a joint venture with Cancer Council NSW, Camperdown, NSW Australia

**Keywords:** Metastatic breast cancer, Advanced breast cancer, Support groups, Psychosocial support, Metastatic survivorship

## Abstract

**Purpose:**

This study explored the value of metastatic breast cancer (MBC) support groups, and factors that affect attendance, from the perspective of people with MBC.

**Methods:**

Semi-structured interviews were conducted with 28 women with MBC (support group attendees *n* = 16; non-attendees *n* = 12) between January 2022 and July 2023. Data were analysed using an inductive approach to thematic analysis.

**Results:**

Three themes were generated: the value of sharing experiential knowledge, spaces for open and honest conversations, and opportunities to find connection and community. These factors were the main reasons that some participants valued, and chose to attend, an MBC support group. Stage-specificity and professional facilitation were identified as important aspects of group structure. Key reasons for non-attendance were concerns about misinformation, confronting the death of group members, and satisfaction with existing support networks.

**Conclusions:**

MBC support groups are beneficial for some people with MBC, providing opportunities to connect with others with the same diagnosis. For others, different forms of peer support such as online forums or one-on-one support may be preferred. We argue that ensuring those with MBC have equal access to the peer support they need will be essential in supporting people to live as well as possible with MBC.

**Implications for Cancer Survivors:**

MBC support groups, if appropriately led, can provide emotional and informational benefits for people with MBC. This research may also have relevance to other metastatic cancers where novel therapies are extending survival, resulting in an emerging cancer population with distinct supportive and survivorship needs.

**Supplementary Information:**

The online version contains supplementary material available at 10.1007/s11764-024-01639-7.

## Introduction

Improvements in treatment for metastatic breast cancer (MBC; cancer that has spread from the breast to one or more distant sites) are driving an increase in the number of patients who are living longer with this diagnosis [1, 2]. The experience of this population has been described as akin to living with a long-term condition, requiring complex ongoing treatment often from different healthcare teams [3]. It is well-established that the psychosocial burden of a breast cancer diagnosis is high, even more so when one is faced with metastatic disease that is incurable and ultimately life-limiting [4–6]. Living long-term with MBC presents an array of physical, psychological, and practical challenges, resulting in diverse, and all too often unmet, supportive care needs [5, 7–13]. In a 2017 survey of Australians with breast cancer, Breast Cancer Network Australia (BCNA) reported that, compared to those with non-metastatic breast cancer, people with MBC had significantly higher information (93% for MBC versus 76% for non-metastatic breast cancer) and support needs (91% for MBC versus 75% for non-metastatic breast cancer) and that those needs were less likely to be met by current support services [11].

Support groups are an established part of cancer supportive care, and their value for people living with cancer in general has long been recognised [14–17]. However, there is evidence that supportive care resources, including support groups, need to be specific to MBC if they are to meet the needs of those living with the illness [18–21]. In an Australian study of women living with MBC, all participants clearly identified a need for specialised, stage-specific support groups for those diagnosed with metastases [21]. The women felt that the majority of support groups were designed for women with early-stage breast cancer (EBC) and consequently left little room for those living with MBC, whose experiences and supportive care needs are very different. This sentiment was echoed in a study from the USA that examined the experiences of women living with MBC in stage-specific versus mixed-stage breast cancer online email-based support groups. Those who had been part of the mixed-stage support groups reported feeling silenced and ostracised, unable to relate to those with EBC [19]. The interviewees felt that the mixed-stage groups created a sense of “them” and “us” that was a barrier to the connection and understanding that is integral to cancer support groups.

The value of stage-specific support groups in metastatic cancer has been the subject of both quantitative and qualitative research since the 1980s. A recent review (2024) summarised the effectiveness of professionally led support groups for people with advanced or metastatic cancer [22], with eight randomised controlled trials undertaken in women with MBC. Almost all of these trials provided strong evidence for a psychosocial benefit of support group participation, including an improvement in mood [23–25], reduction in traumatic stress [24, 26], prevention of depression [26], and improvement in self-reported pain [25, 27]. However, it has been suggested that many of the psychological effects achieved by long-term supportive care interventions in the MBC population cannot be adequately measured using traditional psychometric methods and that qualitative methodologies are more appropriate [28]. Currently, there is a distinct lack of rigorous qualitative research in this area, with recent publications only reporting on small cohort studies of single MBC support groups rather than MBC support groups in general [29, 30]. Additionally, the development of novel treatments such as targeted therapies and immunotherapy has changed the disease trajectory and extended the median survival time of people diagnosed with MBC, potentially shifting how people with MBC value and experience a support group [1]. The historical focus on accepting death, as evidenced in the supportive-expressive group therapy (SEGT) analysed in detail by Spiegel [31–33], may no longer be as appropriate for a new population of people living longer with MBC. Instead, the focus of MBC support groups may need to shift towards including strategies for living as well as possible with MBC [34].

For the purposes of this study, MBC support groups were defined as groups that were stage-specific and conducted face-to-face in real time, either in person or via teleconference. The aim of this study was to examine how people living with MBC perceive MBC support groups, to identify the value of these groups and what factors shaped attendance and/or non-attendance. A better understanding of how MBC support groups are valued and the reasons why people choose to attend (or not) will be critical in informing the design and development of these groups, as well as other supportive care services for people with MBC, in Australia and internationally.

## Methods

### Participants, sampling, and recruitment

Eligible participants were people aged 18 or over with a diagnosis of MBC, who had sufficient command of English to allow them to be interviewed in English and no cognitive impairments. Participants included people who were currently attending, or had previously attended, a stage-specific, synchronous face-to-face (in person or via teleconference) MBC support group as well as those who had never attended an MBC support group. Sampling was purposive in that not all those who contacted us and who met the inclusion criteria were interviewed. Our aim was to interview a range of participants (e.g. different ages, time since metastatic diagnosis, recurrence versus de novo stage IV diagnosis, metropolitan versus regional, support group attendee versus non-attendee) to ensure that we captured the diversity of views on support groups.

Participants were recruited using a variety of strategies, including promotion of the study by support group facilitators, healthcare professionals and consumers using a study flyer, and distribution of promotional material through breast cancer organisations, advocacy groups, and consumer forums, e.g. Breast Cancer Network Australia. Potential participants who contacted the research team were sent a participant information statement and consent form. Completed consent forms were returned to the research team by email.

This broad range of community-based recruitment strategies in combination with purposive sampling ensured participants were included from across Australia and at different stages in their MBC diagnosis. Recruitment and interviews continued until it was judged by the interviewer that sufficient data had been collected to tell a rich, complex, and multi-faceted story that answered the research question [35].

### Data collection

Interviews were semi-structured, using an interview guide that was developed following a review of the literature [9, 16, 36], discussion with the project team, and consultation with consumer representatives living with MBC (Online Resource 1). Interviews were conducted by an experienced qualitative researcher and were undertaken in person, by video teleconference or by telephone between January 2022 and July 2023. Topics included views on support groups, expectations/hopes of a support group, benefits of attending or reasons for not attending, and other forms of support. Probes were used to obtain further information about participants’ experiences and opinions of support groups. Interviews lasted between 45 min to 1.5 h (average 58 min). Detailed field notes were taken immediately after each interview. These notes helped to determine when data adequacy had been reached, i.e. the point at which sufficient rich and complex data had been collected to address the research question [35]. All interviews were audio-recorded, professionally transcribed, and de-identified. Each participant was assigned a pseudonym, and participants were offered an AUD$40 gift voucher as reimbursement for their time.

### Data analysis

This study took a social constructionist approach as we were interested in exploring in-depth the views and experiences of people living with MBC in regard to MBC support groups [37]. Reflexive thematic analysis of the data was undertaken using Braun and Clarke’s guidelines [38, 39], with a primarily inductive and data-driven approach. This involved one author (GM) repeatedly reading the transcripts to identify recurring ideas in the data and initial coding using NVivo software. Codes were then used to develop themes that captured the views of people living with MBC of MBC support groups. After inductive coding of all transcripts, we explored patterns across transcripts, including commonalities and differences between those who did and did not attend a support group. Quotes extracted from the data have been minimally edited, without altering meaning or inference, and ellipses […] indicate that part of the transcript not relevant to the analysis was removed.

### Researcher characteristics and reflexivity

Interviews were carried out by an experienced qualitative researcher who is living with a metastatic breast cancer diagnosis. The impact of the interviewer’s shared health status on study participants was carefully considered, and steps were taken to ensure that the interview focused on the perspective of the participant. For example, the interviewer offered to share her experiences or to answer questions about herself and her diagnosis after the interview rather than during the interview, emphasising that we were interested in the participants’ experiences and perspectives given their unique situation. The interviewer’s in-depth theoretical and personal knowledge was considered a strength of the study, enabling the participants to feel at ease, and for rapport and trust to be developed quickly. However, it was recognised that multiple researcher perspectives were necessary during the analysis and theme development stages. Regular discussion of the theme development process occurred with the whole project team throughout data analysis, with members of the research team bringing a diversity of expertise relevant to the research question, including clinical oncology, health sociology, and qualitative health research methodology. This diversity of knowledge and experience was critical for in-depth analysis of the data and provided a richness to the subsequent interpretation. To ensure power imbalances were kept to a minimum, none of the participants had a pre-existing personal or clinical relationship with any member of the project team. To ensure trustworthiness, we followed O’Brien et al*.* (2014) “Standards for Reporting Qualitative Research” [40].

## Results

Twenty-eight women, aged 34–75 (mean age 56), participated in the study (Table 1). Participants were recruited from metropolitan (61%, *n* = 17/28) and regional (39%, *n* = 11/28) areas across Australia. Of the 28 participants, 22 had access to a stage-specific, face-to-face (in person or via teleconference) MBC support group. Of these 22 women, six reported choosing not to attend the support group. The remaining six participants reported that they were not aware of any metastatic support groups for MBC available in their area. Three overarching themes were derived from the interview data: (1) the value of shared experiential knowledge, (2) spaces for open and honest conversations, and (3) finding connection and community (Table 2). These themes reflect the value that many participants found in their MBC support groups and the factors that encouraged and/or discouraged participants from attending. Key elements that influenced engagement with MBC support groups are detailed in sub-themes, thus providing an insight into the experiences of both support group attendees and non-attendees (Table 2).

**Table 1:** Overview of participant demographics

Characteristics	Number (*n* = 28)
Age (years)	
25–34	1
35–44	1
45–54	6
55–64	15
65 +	5
MBC-specific support group	
Has access and attends	16^a^
Has access and does not attend	6
Does not have access	6
Type of MBC diagnosis	
De novo	14
Recurrence	14
Time since MBC diagnosis (years)	
≤ 1 year	5
2–5 years	14
6–10 years	7
> 10 years	2
Marital status	
Married/partnered	21
Single	2
Widowed	3
Divorced/separated	2
Employment status	
Not working	14
Volunteering	5
Part-time	5
Full-time	4
Level of education	
≤ Year 11	5
Year 12 or equivalent	4
Diploma	5
Undergraduate	6
Postgraduate	8
Residence^b^	
Metropolitan	17
Regional	11

^a^One participant moved states (Tas to Vic) in order to access better treatment and supportive care services

^b^Postcodes were classified using the Australian Statistical Geography Standard (ASGS) remoteness structure (Australian Bureau of Statistics (ABS)) [41]

**Table 2:** Themes and associated sub-themes that support or discourage attendance

**Theme 1: the value of shared experiential knowledge**	
**Supports attendance**	**Discourages attendance**
1. Wanting access to in-person MBC-specific information and advice	1. Having concerns about misinformation
2. Attending increases sense of empowerment in healthcare interactions	2. Wanting to control how much and with whom they share information
3. Giving back to others	
**Theme 2: spaces for open and honest conversations**	
**Supports attendance**	**Discourages attendance**
1. Being prepared to discuss difficult topics	1. Feeling satisfied with existing support networks
2. Feeling relief at not having to burden family and friends	2. Not wanting to be confronted by the deterioration and death of group members
**Theme 3: finding connection and community**	
**Supports attendance**	**Discourages attendance**
1. Finding my tribe: reduced isolation leads to understanding and hope	1. Not wanting to be defined by their MBC diagnosis
2. Feeling disconnected from the early breast cancer survivor narrative/community	2. Wanting to distance themselves from others with MBC

### Theme 1: the value of shared experiential knowledge

All participants (regardless of support group attendance) reported the need for information specific to MBC and placed a high value on experiential knowledge (the knowledge gained through lived experience) that augmented the information provided by their health professionals. Support group attendees reported that MBC support groups provided a space for the personal exchange of this experiential knowledge (Table 3). This included sharing MBC-specific information about treatment (including complementary and alternative therapies), side effects, lifestyle changes, workplace, financial and insurance issues, as well as the impact of their diagnosis on themselves and their families. Participants, like Una (below), indicated that this kind of knowledge would not necessarily be provided by their oncology team, nor would it be discussed in a mixed-stage breast cancer support group. For many participants, access to first-hand experiential knowledge that was MBC-specific was a major reason for attending their support group (Table 3).

"… in terms of information from the support groups … we might look at alternative medicines - things like cannabis oil or what have you. The things that your doctors don’t talk about, that’s really the information that you get." – Una (diagnosed 1 year ago, aged 34, attended MBC group)

**Table 3:** Sub-themes and illustrative quotes for theme 1: the value of shared experiential knowledge

**Supports attendance**	
1. Wanting access to in-person MBC-specific information and advice	“It’s [the support group] made me feel supported in dealing with my drug therapy. It’s informed me of different ways of doing things and supplementary things that might be helpful to my quality of life”. – Gemma (diagnosed 4 years ago, aged 75, attended MBC group) “We’ve all picked up on each other’s lifestyle changes. We’ve discussed things like diet, exercise, complementary medicines [in the support group]”. – Monika (diagnosed 7 years ago, aged 66, attended MBC group) “I think that would be the same things that I’d want to get out of a support group. That you’d get information on planning ahead, advanced care directives, wills, power of attorney, social security, finance, all of that planning ahead sort of stuff”. – Carol (diagnosed 6 years ago, aged 57, did not have access to in-person MBC group) “You pass on information amongst each other [in the support group] that way and also get information on certain drugs that people have been on and how their experience was with it. It’s more targeted, I guess you’d say, to what you need”. – Iris (diagnosed 5 years ago, aged 57, attended MBC group)
2. Attending increases sense of empowerment in healthcare interactions	“So a group helps you articulate what your needs are, which prepares you for better communication, I’m sure, with both your oncologist and your doctor”. – Gemma (diagnosed 4 years ago, aged 75, attended MBC group) “I think that people at [the support group] did inspire me to ask the questions, ask for second opinions, stand up for what I wanted and push for trials and push for other treatments that they maybe weren’t offering”. – Yvette (diagnosed 8 years ago, aged 55, attended MBC group) “I’ve always been a proactive person, but now with the group I realise that if I don’t ask the question, then I’ve only got myself to blame”. – Helina (diagnosed 7 years ago, aged 56, attended MBC group)
3. Giving back to others	“… it was such a relief when I met the [the support group]. I want to be able to offer that to other women who are new and I want to help them like I was helped”. – Yvette (diagnosed 8 years ago, aged 55, attended MBC group) “It did appeal to me, I suppose, that I might be in a group where I could help others, as well”. – Zoe (diagnosed 5 years ago, aged 56, attended MBC group) “I guess to me, the support group is also that to some extent, that you can give back your experience to other people as well as them giving their experience to you … Because I believe that through your shared experience …we can improve outcomes for people”. – Kara (diagnosed 1 year ago, aged 60, attended MBC group)
**Discourages attendance**	
1. Wanting to control how much and with whom they share information	“With the online [groups], I can come and go. If I’ve had a crappy couple of days and … you’re already feeling a little bit stressed and whatever, then I don’t have to go into those groups”. – Sandra (diagnosed 7 years ago, aged 57, did not attend MBC group) “They’re closed [online] groups so that everybody can’t see, because I haven’t shared my diagnosis, only with my family and my closest friends, and my boss. My work colleagues, nobody else. Because I don’t want a pity party”. – Pascale (diagnosed 2 years ago, aged 50, did not attend MBC group) “… the next challenge for me with support groups is that I think because I’ve done so much work as a [profession], the risk if I attend support groups is that I wouldn’t so much get my own support, so much as ending up providing support” – Riki (diagnosed 3 years ago, aged 37, did not attend MBC group)
2. Having concerns about misinformation	“So, in a support group, if the membership were … more into health conspiracy theories and that was how they gained a sense of power and control over their circumstances, it wouldn’t work …” – Riki (diagnosed 3 years ago, aged 37, did not attend MBC group) “There was a real sense … that something needed to change there and that a breast care nurse needed to be involved to strengthen the group and keep things on track a bit, make sure information was being shared that was appropriate, et cetera”. – Monika (diagnosed 7 years ago, aged 66, attended MBC group) “Part of having someone [a professional facilitator] there is monitoring it to identify things like that where they’ll know whether or not that’s a general useful information, or it’s very specific to people”. – Gemma (diagnosed 4 years ago, aged 75, attended MBC group)

Similarly, attendee Monika highlighted the unique nature of the experiential knowledge that was shared in support groups.

"Then there was the dealing with the side-effects, I mean, you can have a sort of discussion with your doctor, but it’s never really the same as having conversations with other women who are experiencing it." – Monika (diagnosed 7 years ago, aged 66, attended MBC group)

Attendees also emphasised that the sharing of experiential knowledge in their support group helped them to feel more empowered when interacting with their healthcare professionals (Table 3). As Monika went on to describe, information and advice from peers gave her more confidence and helped her feel actively engaged in her interactions with her doctor.

"I always come up with a list of questions after the group that I raise at the next clinic appointment … it involves me directly in my own treatment, which is really crucial." – Monika (diagnosed 7 years ago, aged 66, attended MBC group)

The reciprocal nature of shared experiential knowledge within an MBC support group was a key element that encouraged group attendance (Table 3). In addition to learning from others living with MBC, participants acknowledged that the mutual sharing of experiences in a support group was beneficial, as it provided a sense of self-worth and validation. Some support group attendees reported that their group gave them the opportunity to “give back” to others through passing on their own knowledge, often acquired through their participation in the support group. As Farisa describes, she appreciated being able to offer advice and encouragement to those more newly diagnosed, as she understood what it was like to feel isolated with limited options for connecting with others living with MBC.

"I think another thing that I really get out of the group is that I feel like I’m helping the others in a way." – Farisa (diagnosed 2 years ago, aged 49, attended MBC group)

Similar to attendees, participants who chose not to attend a support group also expressed a desire for MBC-specific information about their illness beyond what their medical teams were providing. However, rather than accessing this experiential knowledge from in-person support groups, they instead turned to online resources such as support forums and Facebook groups to fulfil this need. As an example, Sandra, who does not attend an MBC support group, talked about the value of sharing information and hearing positive stories on Facebook, because it gave her more control over when and how she engaged with experiential knowledge. She highlighted that she was able to determine the level of intensity and frequency of her interactions with the MBC community, choosing when to participate and maintaining distance when needed. The ability to “dip in and out” was important to her. This desire to control how much and with whom information is shared was a common reason why participants chose not to attend an in-person, face-to-face MBC support group (Table 3).

"I love the online forum. I love being able to post questions to people who have experience and just get a general consensus of opinions and experiences from people who purport to be going through the same diagnosis as me ... But in a way, it’s at a distance and it’s not necessarily taking on too much emotional baggage from their conditions and their experiences." – Sandra (diagnosed 7 years ago, aged 57, did not attend MBC group)

A small number of participants, all with professional backgrounds in healthcare, who chose not to attend an MBC support group expressed concerns about the possibility that unsubstantiated information and/or misinformation could spread within a support group (Table 3). For these participants, this fear of misinformation proved to be a barrier to support group attendance. These apprehensions were illustrated by Sandra:

"The one fear I have as well on a lot of these support groups is people sharing incorrect information, people not quite understanding what they’ve been told and then passing that on as gospel." – Sandra (diagnosed 7 years ago, aged 57, did not attend MBC group)

Of the participants who did not attend an MBC support group, half did not have access to an in-person group. Almost all of these women expressed a desire for access to an MBC support group, and similarly to attendees, all reported a need for practical information and advice specific to MBC.

"I just think there’s so much I could learn from other people who have metastatic breast cancer." – Carol (diagnosed 6 years ago, aged 57, did not have access to in-person MBC group)

Interestingly, this group of women did not appear to access online support forums or were more dissatisfied with the online format than those women with access to a group who chose not to attend. One participant who did not have access to an MBC support group, Riki, was an exception to this—she relied heavily on online Facebook groups for information and support. Similar to Sandra above, these types of groups allowed her to maintain distance and control over what information she accessed and when.

A shared view across both attendees and non-attendees was that an MBC support group should be professionally led, rather than peer-led. Women felt that a professional facilitator would have access to resources that a peer leader would not, for example, guest speakers, current medical advice, and information about referral pathways. This type of group facilitation could also provide more protection against the spread of misinformation that was concerning for some participants. Furthermore, a professional facilitator would have the skills and experience to manage the complex psychosocial needs of a diverse group of women.

"I really value just … having [the breast care nurse] guide you and guide the group, guide the discussion, provide information, provide people with contacts that they might need; that’s not such a safe thing to do in an unregulated group." – Gemma (diagnosed 4 years ago, aged 75, attended MBC group)

### Theme 2: spaces for open and honest conversations

A second prominent theme was the importance of having dedicated spaces for those with MBC to have open, authentic interactions with peers (others living with MBC). Those who attended a support group described this as a place where they felt truly safe to talk, without concern for the emotional burden on others. This theme was characterised by two prominent sub-themes that captured the value that attendees found in having a safe space to talk, and the reasons that encouraged their continued attendance (Table 4).

**Table 4:** Sub-themes and illustrative quotes for theme 2: spaces for open and honest conversations

**Supports attendance**	
1. Being prepared to discuss difficult topics	“But what is a good death? Being able to talk freely about things like that would just be such a relief”. – Angela (diagnosed 1 year ago, aged 59, did not have access to MBC group) “We also have laughs [in the support group], and … we laughed about silly things like how we might design our coffin … we don’t feel threatened. We can talk about anything”. – Bianca (diagnosed 2 years ago, aged 63, attended MBC group) “The other thing, I’ve seen a lot of women over the years pass away with MBC from the [support group]. It’s really demystified the death process”. – Yvette (diagnosed 8 years ago, aged 55, attended MBC group) “The main thing is that when you do have people facing death, you need the facilitators to help the rest of the group cope with that and get through it. They really come into their own when there is a trauma or drama going on or someone’s at the end of life. They provide that support and cohesion and help us make sense of what we’re feeling”. – Yvette (diagnosed 8 years ago, aged 55, attended MBC group)
2. Feeling relief at not having to burden family and friends	“It [the support group] helps because you’ve got an outlet to speak of these things. Even with your partner, when they’re around, you really don’t want to bring up the heavy stuff with them”. – Iris (diagnosed 5 years ago, aged 57, attended MBC group) “It’s like you’re frightened, and you want to tell somebody something, sometimes it’s better to tell the group than put that fear on to my husband or my daughter”. **–** Olivia (diagnosed 4 years ago, aged 64, attended MBC group) “I just can’t tell you what it means to be in a room where you know you’re safe and you can say it sucks. That is extremely important”. – Nora (diagnosed 4 years ago, aged 67, attended MBC group) “It’s nice that you can go to your support group, you don’t have to be positive. You can just be real. You can have a cry. You can let it all out. They just get it”. – Una (diagnosed 1 year ago, aged 34, attended MBC group)
**Discourages attendance**	
1. Feeling satisfied with existing support networks	“I didn’t think, oh God I need to have a group thing because I sort of got that feeling, the group feeling from … the exercise classes of mine … So, I guess my needs were covered there” – Agatha (diagnosed 3 years ago, aged 66, did not attend MBC group) “Yeah, yeah, I would happily and openly talk to all of them [friends and family] about my deepest, darkest fears. My husband is amazing. He’s like a little mop. He just soaks it all up”. – Sandra (diagnosed 7 years ago, aged 57, did not attend MBC group) “I had a metastatic breast cancer nurse through the McGrath Foundation. It was amazing. Incredibly supportive, incredibly helpful” – Riki (diagnosed 3 years ago, aged 37, did not attend MBC group)
2. Not wanting to be confronted by the deterioration and death of group members	“I must admit it doesn’t appeal to me to go to a metastatic only group just from that point of view. I’d be scared of seeing people who were close to death”. – Janni (diagnosed 22 years ago, aged 60, did not attend MBC group) “Getting to know the whole of that person in person and then having them die is even more difficult to deal with, so I think my gut instinct would be to not go to anything that’s up close and personal”. – Sandra (diagnosed 7 years ago, aged 57, did not attend MBC group) “It both relieves my anxiety and sometimes creates some. I don’t mind being confronted by death, but it probably takes up more head space than it did before I joined the group”. – Zoe (diagnosed 5 years ago, aged 56, attended MBC group)

Firstly, many women positioned their support group as providing a space for conversations about difficult topics that might otherwise have been avoided with family, friends, or doctors. These included discussions about prognosis, treatment side effects, sex and marital relationships, and death and dying (Table 4).

"Because people don’t really want to know what your medical condition is, and where you’re up to with it … So there’s not many places or many people in whom you can confide your true situation. A group that’s set up for that purpose is a huge boost." – Gemma (diagnosed 4 years ago, aged 75, attended MBC group)

Open discussion within their group about weightier topics such as mortality and death was generally viewed by attendees as valuable. As Bianca describes, “it’s just showing that someone that’s knowing that their journey is coming to an end can be still positive … that gives me courage”. For some, getting to know women who subsequently died from MBC demystified the process of dying and ameliorated some of the fear associated with what was a previously unknown experience.

Secondly, many who attended a support group stated that being able to have these more difficult conversations with their group lifted an emotional burden on family and friends. Relief of a perceived burden on loved ones was a common reason for both seeking out an MBC support group, and regular attendance (Table 4).

"You see the support of the group when there’s something you’re … thinking about, and you don’t feel that you can bring this topic up within your own friend group or your own family." – Helina (diagnosed 7 years ago, aged 56, attended MBC group)

Participants, both attendees and non-attendees, described feeling the need to present a happier façade around family and friends, and apprehension about being regarded as too negative often prevented them from sharing greater concerns, particularly around the incurable nature of MBC, with loved ones. This mindset existed even when women reported that they felt well-supported by their family and friends. The MBC support group provided a protected space where participants could openly discuss their fears with other women who understood the challenges of living with MBC.

"I just wanted to meet people where I could talk openly and safely about metastatic cancer without anybody crying or losing the plot…" – Nora (diagnosed 4 years ago, aged 67, attended MBC group)

For Larissa, who did not have access to a metastatic support group in her area but planned on undertaking a 4-h round trip to attend one, the most important aspect of a group was relieving a perceived burden on her family:

"I think it [the support group] will take a burden off my husband, that’s my biggest thing." – Larissa (diagnosed 1 year ago, aged 62, did not have access to in-person MBC group)

In contrast, whilst non-attendees still valued authentic interactions with others about their experience of MBC, they felt that they currently had other adequate social support networks in their lives (Table 4). Women who chose not to attend a support group for MBC felt that they were able to discuss their concerns and emotions openly within their close relationships and did not experience negative reactions to disclosing their more serious fears. Others reported that regular involvement in other activity-based groups (e.g. exercise classes) provided them with an adequate network of support. Several non-attendees also described the high value of their metastatic breast care nurse. These external relationships appeared to alleviate the need for an MBC support group for some participants. Barbara, however, found that the chronicity of an MBC diagnosis meant that the extended support she needed could not feasibly be provided by family and friends. This resulted in her seeking out an MBC support group months after her initial diagnosis.

"So, the first couple of months, I actually [had] a lot of family support and that was fine. That was meeting all my physical, emotional, and household needs … and then they all left and progressively I just fell into a hole, emotionally." – Barbara (diagnosed 2 years ago, aged 50, attended MBC group)

This experience was shared by Queenie, who articulated how the nature of an MBC diagnosis could be misunderstood, resulting in the need for additional long-term support. Whilst she had yet to attend, she was in the process of seeking out an MBC group to fill this supportive care gap.

"People see me and think I’m well and they sort of forget that I’m sick and not that I want them to be remembering that I’m sick but I’m probably carrying the burden of it a bit more on my own than I was at the beginning. Because initially, I think everyone, including me, thought I was going to be dead within a few years so people kind of rallied around. … I think I probably need a bit more of that where I need to be sitting there with other people who are having the same kind of crap going on…" – Queenie (diagnosed 6 years ago, aged 56, did not attend MBC support group)

For other participants, a barrier to attendance was trepidation that an MBC support group was *not* a safe space. Women who chose not to attend a support group described their fears of the emotional negativity that they attributed to metastatic support groups, in particular not wanting to be confronted with the deterioration and death of other group members (Table 4). Of note, participants who were particularly concerned about the emotional challenges of an MBC support group had not previously attended this type of group before; their decision not to attend appeared to be shaped by their assumptions and expectations of such a group, which were often negative.

"I guess that’s the danger of getting involved in support groups. It’s probably something that turns me off getting too involved, because I don’t want someone dragging my brain to those negative places…" – Sandra (diagnosed 7 years ago, aged 57, did not attend MBC group)

Non-attendees spoke about their worries that seeing disease progression in other members would be too confronting. The idea of connecting with other people living with MBC who might then die was particularly distressing to some participants. Janni, who had previously attended a mixed-stage group after she was diagnosed with MBC but has never attended a metastatic group, described her feeling that: “The trouble with a metastatic disease group I feel is that it’s pretty doom and gloom, you know”.

Other women reported that the thought of joining a support group in the early days of their MBC diagnosis was difficult to comprehend, because they needed time to process their own emotions and did not feel as if they had the emotional capacity to hear about the challenges other women were facing. The thought of having to confront emotions such as grief, loss, and fear experienced by other women was a distinct barrier that prevented these women from seeking out and attending an MBC support group.

"I was trying to understand it myself before I could then go and talk about it with other people and be present and acknowledge what they’re going through as well as what I’m going through." – Tilly (diagnosed 1 year ago, aged 49, attended MBC group)

However, for some of these women, like Tilly (above), their ideas about MBC support groups shifted over time, and as they adjusted to living with metastases. Tilly eventually sought out the support of an MBC group, explaining that: “I kind of need someone to be able to chat to that I can have a really deep and raw conversation with”. Many attendees acknowledged that whilst there were challenging aspects of participating in an MBC support group, these were outweighed by the associated benefits, including opportunities for connection and emotional support.

"Yes, it’s hard and it’s challenging and people are sick … You can’t help, of course, reflect on your own situation when you see other members dying, but I just really think the pros outweigh the cons." – Yvette (diagnosed 8 years ago, aged 55, attended MBC group)

Similar to theme 1, where a professional facilitator was valued for being able to manage concerns about misinformation, the presence of a trained facilitator was seen as key to ensuring that support groups were safe spaces for women.

"I think sometimes we’re disclosing very personal … emotional issues in the group. You can’t just open a can of worms and then leave it sitting there. I think you have to be able to deal with what comes up. You need a professional to be able to guide us through that." – Yvette (diagnosed 8 years ago, aged 55, attended MBC group)

### Theme 3: finding connection and community

Most participants, both attendees and non-attendees, reported not knowing anyone else living with MBC when initially diagnosed and frequently felt extremely isolated. Participants often described the frustration and loneliness they felt when people who did not have MBC did not understand or recognise the challenges they were facing. Connection with other women in an MBC support group reduced feelings of isolation for many attendees. This was a common factor that encouraged attendance at an MBC support group (Table 5). Barbara explained that she sought out a metastatic support group because: “I think fundamentally I just wanted to connect with other people with metastatic breast cancer because I was feeling so alone with it”.

**Table 5:** Sub-themes and illustrative quotes for theme 3: finding connection and community

**Supports attendance**	
1. Finding my tribe: reduced isolation leads to understanding and hope	“There was that sense of being alone, then I found my tribe”. – Larissa (diagnosed 1 year ago, aged 62, did not have access to MBC group) “I think from the support group it would be just being with other people, hearing them talk. Yeah, feeling like you belong to something I think”. – Carol (diagnosed 6 years ago, aged 57, did not have access to MBC group) “What was it that I was actually seeking by seeking out support groups? I think fundamentally, it's just connection with other people with my condition”. – Barbara (diagnosed 2 years ago, aged 50, attended MBC group) “Never underestimate that role of mutual support, I think, it’s been vital for me”. – Monika (diagnosed 7 years ago, aged 66, attended MBC group) “I think the group has helped me in terms of giving me hope and inspiration from the other women. Just a burden shared is not as heavy, I think”. – Yvette (diagnosed 8 years ago, aged 55, attended MBC group)
2. Feeling disconnected from the early breast cancer survivor narrative/community	“I feel so left out, I feel like I can’t run around in my pink t-shirt waving my arms and singing I’m a survivor”. – Gemma (diagnosed 4 years ago, aged 75, attended MBC group) “There is no happy clappy and pink ribbons when you have a stage 4 diagnosis” – Sandra (diagnosed 7 years ago, aged 57, did not attend MBC group) “To be honest, I’m in a few online groups, and I’ve stuck specifically with the MBC stuff, because I was sick of hearing people with Stage 1 moaning and groaning …” – Vera (diagnosed 2 years ago, aged 50, attended MBC group) “I think there’s such different needs and different outcomes and different trajectories for the two different groups that it can almost be harmful, I think, to put early breast cancer patients with the late stage or vice versa because, yeah, the needs are just so different. Honestly we’re the early breast cancer patients’ worst nightmare, aren’t we?” – Yvette (diagnosed 8 years ago, aged 55, attended MBC group)
**Discourages attendance**	
1. Not wanting to be defined by their MBC diagnosis	“It’s not that I try and ignore my diagnosis. I’m very well aware of it. But … I don’t like dwelling on it”. – Sandra (diagnosed 7 years ago, aged 57, did not attend MBC group) “For me, my identity as a [profession] is much more important to me than my identity as a cancer patient”. – Riki (diagnosed 3 years ago, aged 37, did not attend MBC group) “So, when I was seeking support and thinking about the effects of cancer on identity, it’s often more about how it affects my professional self, but also my physical ability and stuff. But not so much how cancer affects my sense of who I am as a person”. – Riki (diagnosed 3 years ago, aged 37, did not attend MBC group)
2. Wanting to distance themselves from others with MBC	“These groups are not for everybody but there are times when you do feel like, I don’t want to hear this again, I just want to have a life separate from cancer for a few weeks or whatever”. – Monika (diagnosed 7 years ago, aged 66, attended MBC group) “I feel like I don’t belong in that [metastatic] group yet, I will when it does come back but I don’t want to be – I’d rather be in the group that’s doing yoga or doing exercise classes and just getting on with life”. – Agatha (diagnosed 3 years ago, aged 66, did not attend MBC group) “Again I just want to get on with living. When the day comes that this beast starts creating havoc again, well, then I’ll just have to deal with it. But while it’s at bay and I can get on with living life, that’s what I want to focus on”. – Sandra (diagnosed 7 years ago, aged 57, did not attend MBC group)

Participants described the desire to find one’s “tribe” or “sisterhood” and expressed an overwhelming need to connect with other people with MBC (Table 5).

"I needed a group that were experiencing the same emotional spectrum that I was experiencing, and often taking similar or same drugs, some of them working with the same oncologist. So it was like being with people who understood…" – Gemma (diagnosed 4 years ago, aged 75, attended MBC group)

MBC support groups were a source of connection and understanding that only other women living with MBC could provide. This feeling was distinctly different from the support participants received from their medical team, family, or friends and was expressed by both support group attendees and non-attendees.

"It doesn’t matter how good the care is, at the end of the day, you do need support from people of your own, and that’s why I call them [support group] my tribe." – Bianca (diagnosed 2 years ago, aged 63, attended MBC group)

Support groups were also described as a much-needed source of hope, particularly if the women could see others who had been living well with MBC for many years.

Additionally, participants strongly felt that the experience of an MBC diagnosis was distinct from early-stage breast cancer (EBC). This sense of separation from those with EBC was reflected in the desire of many women for MBC support groups, as they sought connection with women who could fully understand and appreciate the distinctness of their experience. Many women highlighted the disconnect they felt with the mainstream breast cancer community and the survivorship narrative that is often associated with EBC (Table 5). Bianca, who had previously attended a mixed-stage support group after her diagnosis of MBC (but now attends an MBC group), felt that the other women with EBC did not know how to relate to her, which meant that she did not feel truly included in the group.

"I felt as if I was still a bit of an outsider [in the mixed-stage support group]… I just think that maybe that it was a bit threatening for them, and they didn’t know how to deal with my issue." – Bianca (diagnosed 2 years ago, aged 63, attended MBC group)

This lack of connection often formed the basis for continued attendance at an MBC support group. However, not all participants felt this way—Janni attended a mixed-stage support group for young women when she was initially diagnosed at age 37 and was the only one with MBC. She did not experience the same marginalisation as Bianca (above) because she was able to relate to the other women with EBC on a different level, as a young single mother.

Amongst participants who chose not to attend an MBC support group, the desire to connect with others living with MBC was less pronounced, a key reason being that they did not want their diagnosis to define them, and they did not want to be constantly immersed in the cancer world (Table 5). Sandra, who chooses not to attend a support group, described her feeling that: “The more involved you get in groups like that, the more they can consume your life … I don’t want to give it air. It’s got enough of my life already”. Women who did not attend a support group were also commonly still working, either full- or part-time. Additionally, non-attendees were more likely to report minimal impact of their MBC diagnosis on day-to-day life, as they were medically stable and felt physically well. These factors also appeared to reduce the need for an MBC support group.

"I think I get by in coping with this because I’m really not affected by it very much medically speaking at all." – Agatha (diagnosed 3 years ago, aged 66, did not attend MBC group)

One participant who did not have access to an MBC support group, Riki, expressed a similar sentiment and described how: “Cancer is something I have to deal with, but it’s not so much my identity”. This perspective was influenced by her individual illness experience and personal circumstances—she felt unable to relate to other women with MBC due to her younger age, professional background, and because she did not want to have children. As a result, she preferred to seek support in an online or one-on-one format.

In contrast to other non-attendees, the majority of participants who did not have access to an in-person MBC support group expressed a strong need to connect with other people living with MBC. Larissa, for example, described the feeling of being marginalised by the mainstream breast cancer community: “We’re the wrong shade of pink”. These women felt isolated and emphasised their desire for community, belonging, validation, and normalisation of their experience through connecting with others with MBC.

"I think they [support groups] would be fantastic. I’ve never needed one before but I really feel I gravitate towards other women at the moment because non-cancer people don’t get it." – Angela (diagnosed 1 year ago, aged 59, did not have access to in-person MBC group)

Several women indicated that they thought access to one-on-one peer support would have been useful, particularly in the early stages of their diagnosis. They felt that talking to another individual was less confronting than attending a support group and would have preferred one-on-one peer support to attending an MBC support group.

"I think a one-on-one match up would be probably a good way to go about things rather than any kind of a group thing for those early days." – Sandra (diagnosed 7 years ago, aged 57, did not attend MBC group)

"… it would have been good on a one to one. I think maybe if I wasn’t ready to go into a group where possibly it would have been bit more confronting." – Queenie (diagnosed 6 years ago, aged 56, did not attend MBC support group)

## Discussion

This Australian study is one of the first to provide a rich, in-depth account of the experiences and perspectives of women living with MBC of stage-specific, face-to-face (in person or via teleconference) MBC support groups. The study demonstrates that whilst participants appreciated the care they received from their medical team, many also desired connection to others with MBC. This is consistent with previous research about the experience of living with MBC [6, 42] and highlights the importance of supportive, not just medical, care. For those who attended an MBC support group, the key benefits included the sharing of experiential knowledge, the opportunity to talk openly about difficult topics, and the sense of community with others living with MBC. The role of professional facilitators was particularly valued, as their presence created a safe space to address sensitive topics, such as anxiety about the future and fear of disease progression, that may have otherwise been avoided and to address misinformation. The main factors discouraging attendance were concerns about the regulation of information sharing, the presence of adequate external support, and not wanting to confront the deterioration and death of other group members, as well as a reluctance to engage in cancer-related discourse.

In this study, both attendees and non-attendees reported the need for information specific to MBC and placed a high value on experiential knowledge. For attendees, their support group was perceived as a valuable source of first-hand disease-specific information, an often unmet need for this population [7, 8, 21, 43]. The association of experiential knowledge to notions of empowerment and agency was prominent. This has previously been identified in the literature; Vilhauer et al*.* (2009) reported that women who participated in an online asynchronous email MBC support group felt that their group gave them the confidence to seek out better medical care and initiate discussions with their healthcare providers, making them more active in their treatment [36]. In another study of online support groups for people with breast cancer, fibromyalgia, or arthritis, almost all participants reported that participating in an online group increased their confidence in their interactions with their physicians [44]. Our study demonstrates that participants who attended an in-person MBC support group experienced similar improvements in confidence, assertiveness, and feelings of empowerment, which was directly attributed to shared experiential knowledge.

This study also illustrates how MBC support groups provide a rare avenue for those living with MBC to connect with others with the same diagnosis. Those who felt that others misunderstood their identity as a person living with MBC desired connection with others with shared experiences. This was an important factor that encouraged support group attendance. The sense of connection within an MBC support group significantly reduced isolation for participants and contributed to a feeling of belonging within a new-found community. Feelings of isolation after being diagnosed with MBC were frequently described in this study and are commonly reported in the literature [29, 42, 43]. Whilst family and friends provided good support, women were often reluctant to share their deeper fears and concerns with them. A perceived need to protect loved ones from the reality of their diagnosis is a common psychological burden for those living with MBC [4, 5, 42], and this study, amongst others, highlights the importance of having a place where this burden can be relieved [19, 29, 33]. Participants also expressed the feeling that they had limited opportunities to meet other women with MBC outside of a support group. This sense of isolation is perhaps amplified by the current treatments available for MBC, as women are increasingly being treated with oral therapies, which limits opportunities to connect with the hospital system and with others living with the same diagnosis. These same treatments have prolonged the lifespan of those with MBC and thus changed the nature of psychosocial support required to live well with MBC [45]. The chronicity of an MBC diagnosis can prove difficult for family and friends who, as described in our study, may struggle to provide long-term psychosocial support. These factors emphasise the importance of increasing access to MBC support groups, amongst other supportive care options.

However, women’s experiences and perspectives on MBC support groups were not homogenous. Participants who chose not to attend a support group, whilst not in denial about their diagnosis, appeared to want to avoid reminders of what might lie ahead, for example, disease progression. They often retained more ties to their life pre-diagnosis that kept them busy and occupied, such as work or exercise classes, which perhaps served as a diversion from their diagnosis and acted as barriers to support group attendance. This is consistent with previous studies that explored strategies people employed for coping with living with MBC, including avoidance of triggers such as talking about death or dying, distraction with exercise or social company, and continuing to work [5, 34]. This allowed women to maintain a sense of normalcy, which our study suggests may reduce the need for an MBC support group. It is also possible that non-attendees are at a different stage of adaption to their MBC diagnosis, in a process described by Davies and Sque (2002) where patients must come to terms with a new, altered identity and accept that they cannot return to their pre-cancer self [6]. Non-attendees may be more reluctant to engage with face-to-face support groups if they have not completely accepted their own changed identity.

Other significant barriers to support group attendance reported in this study were centred around the perception of MBC support groups. Many non-attendees had negative pre-conceived ideas of the support group model of care, including concerns about spreading misinformation and the potential emotional challenges of a metastatic-specific group, such as confronting the deterioration and death of other group members. These issues have been discussed previously in other studies on support groups for MBC [19, 36] and have also been reported amongst EBC survivors [46]. Interestingly, in our study, the participants who consciously chose not to attend a support group had no previous experience with face-to-face MBC groups. Their decisions not to attend appeared to be shaped by the expectations and values they held about cancer survivorship, as well as the perceived nature of MBC support groups. These women did not identify with what they imagined a support group attendee embodied and had pre-conceived ideas and assumptions about the type of discussions that would take place in an MBC group.

Instead of a face-to-face MBC support group, women in our study who chose not to attend a group often utilised online MBC forums for their information and support needs. This decision to engage in online supportive care communities rather than face-to-face MBC support groups may stem from a desire to remain in control of the information consumed. A digital space affords more control over how and when women with MBC choose to engage with their support network and the type of information they access [47]. In contrast, in a face-to-face setting, the group dynamics, other attendees, and topics of discussion are perhaps perceived as unpredictable factors that women feel they have less control over. Studies by Vilhauer suggest that an online or computer-mediated format for MBC support groups can provide a more acceptable format for some women, particularly those who have been more recently diagnosed and may still be struggling to come to terms with their prognosis [36, 47]. Online support groups allowed women to receive the support they needed on their own terms and provided a shield from the distress of others when required. The results from our study also suggest that a one-on-one model of peer support can be preferrable for similar reasons. One-on-one peer support has been established as an effective model of support in EBC patients [48–50], and our study indicates that these benefits could extend to people living with MBC as well. Overall, it is apparent that a suite of peer support options from which women can choose from in accordance with their specific circumstances (e.g. time since diagnosis, age, MBC subtype, type of treatment) and social context is important to ensure women are supported to live as well as possible with MBC.

Our study identified several key structural features of an MBC support group that influenced the acceptability of a group and impacted participants’ willingness to attend. These include professional facilitation and stage-specificity. The responses of participants who had attended an MBC support group suggest that the concerns of some non-attendees around the discussions and information shared within a group could be appropriately addressed by professional facilitation, which has been identified as valuable in this population [29, 47]*.* The value of a professional facilitator was evident throughout the study, as participants described how they were able to moderate information sharing within a group (theme 1), create a protected space for emotional conversations (theme 2), and foster a sense of community within the group (theme 3). Women who attended an MBC support group emphasised the importance of their professional facilitator in managing group dynamics and creating a safe space for open discussions.

Stage-specificity was another integral structural feature of support groups for MBC identified in this study. Participants reported a disconnect from those living with EBC and a need for supportive care resources that were specific to MBC. The recovery narrative of general breast cancer support groups is typically one that emphasises the positive features of being a “survivor” [51]. This does not reflect the reality of women living with MBC and was, as a result, often viewed as unacceptable by the participants in our study. This sentiment has been previously discussed in the literature—women with MBC do not feel included in online mixed-stage groups and struggle to relate to the mainstream breast cancer community [19, 52]. Interestingly, one Australian study in cancer support group participants found that the majority (93%) of respondents thought that support groups should not be separated by prognosis [53]. However, only 27% of respondents had advanced disease and people who did not attend or who had withdrawn from a support group were excluded from the study, so the views of those unsatisfied with the group format were not represented. Consistent with previous research recommending the stage-specificity of support groups [15, 18, 19, 21, 44], our data strongly suggest that support groups for MBC should be stage-specific in order to be of value to those living with the disease.

A strength of this qualitative study relates to the rigour of the study, in particular the completeness of the data collection, the adequacy of the sample in terms of its ability to supply all the information needed for a comprehensive analysis, and the diverse perspectives brought to the analysis including lived experience, and methodological and content expertise. Inclusion of people who attended, chose not to attend, or who had no access to an MBC support group generated rich, complex data. The subsequent analysis went beyond the descriptive and explicit to interrogate the implicit (latent) meaning [35] and addressed all of the variation and complexity observed [54]. This study and its findings fill an important research and knowledge gap. There is a paucity of recent published research in this area, as highlighted by a 2024 scoping review looking at evidence for the effectiveness of professionally led support groups for people with advanced or metastatic cancer [22]. Only 2 out of 19 identified studies in this field were published in the last 10 years, only one of which was relevant to MBC [30]. Of the 9 RCTs conducted on MBC support groups, all are now over 15 years old, and 7 are over 20 years old [24–26, 28, 31, 55–58]. Many other qualitative research papers in this field are also 10–15 years old [19, 33, 36, 47, 59–61]. Another strength of this study is that, whilst limited to Australian participants, the findings are applicable internationally given that similar issues such as isolation and lack of targeted support have been identified in MBC populations overseas [19, 29, 34].

Tong’s COREQ criteria provided a framework for reporting the methods used in this study, in order to improve the transparency of data collection and analysis [62]. Following these criteria, this study has several limitations. Firstly, participant demographics, whilst varied in terms of age, location, and time since MBC diagnosis, did not capture information on ethnicity or socio-economic background. As such, their views may not reflect the experiences of all women living with MBC, limiting the transferability of our findings beyond this cohort. Only one researcher (GM) was responsible for initial data coding; however, theme creation was undertaken with input from the entire research team in order to utilise the breadth of experience available. Additionally, whilst the data were analysed together and were not separated according to support group attendance, each piece of data was traceable to its source and clearly took into account whether the participant attended a support group or not. Our project team also included three consumer representatives with MBC, who provided their input into the interpretation of our findings.

## Conclusion

The findings from our study demonstrate that many of the unmet needs of those living with MBC [8, 11–13] could potentially be met by MBC support groups. These groups, if properly implemented, have the potential to provide long-term support to women over the course of their diagnosis. How support groups are valued and decisions to attend or not are shaped by a constellation of factors, including personal circumstances, pre-existing support networks, and illness trajectory. The barriers to attendance at a support group identified in this study highlight that MBC support groups are not appropriate for all women at all stages of their MBC journey. This reinforces the need for different modes of delivery of supportive care resources, such as one-on-one peer support, online support groups, and specialised metastatic breast care nurses. Further research should be focused on identifying systems-level barriers to the implementation and sustainability of MBC support groups, as well as understanding the perspectives of group facilitators, to inform the development of these groups in Australia.

## Supplementary Information

Below is the link to the electronic supplementary material.

Supplementary file1 (DOCX 17 KB)

## Data Availability

The de-identified datasets generated during and/or analysed during the current study are available from the corresponding author on reasonable request.
