# Supplementary material for: Finding my tribe: a qualitative interview study of how people living with metastatic breast cancer perceive support groups
Source: J Cancer Surviv. 2024 Jul 24;20(1):107–22. doi: 10.1007/s11764-024-01639-7 (PMC12906536; doi:10.1007/s11764-024-01639-7)
Supplement: Supplementary file 1 — Supplementary file1 (DOCX 17 KB) [file 11764_2024_1639_MOESM1_ESM.docx]

TITLE: Finding my tribe: a qualitative interview study of how people living with metastatic breast cancer perceive metastatic breast cancer support groups

Journal of Cancer Survivorship

Grace M Mackie^1^, Frances Boyle^1,2^, Sophie Lewis^1^, Andrea L Smith^3,+^

+ Corresponding author: Dr Andrea L Smith, Rm 111b, Edward Ford Building (A27), University of Sydney, Camperdown NSW 2006, Australia. Telephone: 0405622525; email: [andrea.smith@sydney.edu.au](mailto:andrea.smith@sydney.edu.au)

1. Faculty of Medicine and Health, University of Sydney, NSW
2. Mater Hospital, North Sydney, NSW
3. The Daffodil Centre, University of Sydney, a joint venture with Cancer Council NSW, NSW

Supplementary table 1: Semi-structured interview guide

| **Support group attendees** | **Support group non-attendees** |
| --- | --- |
| What are your views about support groups in general? | What are your views about support groups in general? |
| How did you come to be part of a support group? | What are your thoughts on support groups for people with cancer? |
| What did you hope to gain from joining the group? | What experience have you personally of support groups? |
| What do you get out of participating in this support group (that you don’t get elsewhere)? | Do you know of any support groups that you could attend if you wanted to? |
| How has being part of this group influenced how you cope with your diagnosis? | Can you tell me a bit about why you don’t attend a support group? |
| Are there any social or supportive activities that happen outside of formal group sessions? | What would a support group have to look like to make it something you would want to go to? |
| How does participating in the group interact with your other support networks? | Where do you get your support from? |
| Have you found anything difficult/challenging about being part of the support group? | What other forms of support would you like to have better access to? |
| If you could change anything about the group, what would it be? |  |
| Why do you continue to attend the group? |  |
